# A novel signature based on pyroptosis-related genes for predicting prognosis and treatment response in prostate cancer patients

**DOI:** 10.3389/fgene.2022.1006151

**Published:** 2022-10-27

**Authors:** Xi Xiao, Jianpeng Li, Shun Wan, Mingzhe Wu, Zonglin Li, Junqiang Tian, Jun Mi

**Affiliations:** ^1^ Department of Urology, Lanzhou University Second Hospital, Lanzhou, China; ^2^ Key Laboratory of Gansu Province for Urological Diseases, Lanzhou, China

**Keywords:** prostate cancer, pyroptosis, tumor microenvironment, immune checkpoint inhibitor, treatment response

## Abstract

**Background:** Pyroptosis is a form of programmed cell death accompanied by specific inflammatory and immune responses, and it is closely related to the occurrence and progression of various cancers. However, the roles of pyroptosis-related genes (PRGs) in the prognosis, treatment response, and tumor microenvironment (TME) of prostate cancer (PCa) remain to be investigated.

**Methods:** The mRNA expression data and clinical information of PCa patients were obtained from the Cancer Genome Atlas database (TCGA) and the cBioPortal for Cancer Genomics website, and the 52 PRGs were obtained from the published papers. The univariate, multivariate, and LASSO Cox regression algorithms were used to obtain prognostic hub PRGs. Meanwhile, qRT-PCR was used to validate the expression of hub genes between PCa lines and normal prostate epithelial cell lines. We then constructed and validated a risk model associated with the patient’s disease-free survival (DFS). Finally, the relationships between risk score and clinicopathological characteristics, tumor immune microenvironment, and drug treatment response of PCa were systematically analyzed.

**Results:** A prognostic risk model was constructed with 6 hub PRGs (CHMP4C, GSDMB, NOD2, PLCG1, CYCS, GPX4), and patients were divided into high and low-risk groups by median risk score. The risk score was confirmed to be an independent prognostic factor for PCa in both the training and external validation sets. Patients in the high-risk group had a worse prognosis than those in the low-risk group, and they had more increased somatic mutations, higher immune cell infiltration and higher expression of immune checkpoint-related genes. Moreover, they were more sensitive to cell cycle-related chemotherapeutic drugs and might be more responsive to immunotherapy.

**Conclusion:** In our study, pyroptosis played a significant role in the management of the prognosis and tumor microenvironment of PCa. Meanwhile, the established model might help to develop more effective individual treatment strategies.

## Introduction

Prostate cancer (PCa) is the world’s second most frequent male malignancy, and it causes significant health problems for men (Sung et al., 2021). In the United States, the number of new cases in 2021 is expected to be around 248,530, with around 34,130 fatalities (Siegel et al., 2021). Although PCa has a higher overall survival rate than some other cancers, it has a very high recurrence rate. Many patients will experience disease progression and eventually develop castration-resistant prostate cancer (CRPC), which is incurable and may become drug resistant (De Angelis et al., 2014; Fujita and Nonomura, 2019; Howard et al., 2019). Certainly, Individualized chemotherapy and immunotherapy have a good prospect of promise for improving the prognosis of PCa patients (Dudzinski et al., 2019). However, immunotherapy has a low response rate in unselected PCa patients (Sandhu et al., 2021). Fortunately, genetic testing is becoming increasingly beneficial for treating patients with PCa (Merseburger et al., 2021). That is, because identification of target genes can guide patients to assess cancer risk, conduct, precision medicine treatment (such as individualized chemotherapy and immunotherapy), and manage disease prognosis (Giri et al., 2018). Therefore, further studies into the molecular mechanisms of PCa, and the development of effective biomarkers, are required to improve patient prognosis and quality of life.

Pyroptosis is a novel mechanism of programmed cell death triggered by some inflammasomes. Pyroptosis causes cell swelling, plasma membrane lysis, chromatin breakage, and cell content release *via* particular pathways, resulting in a potent inflammatory response. Pyroptotic cells are unique in maintaining nuclear integrity (Shi et al., 2015; Ding et al., 2016; Kovacs and Miao, 2017; Fang et al., 2020). Generally, there are three pathways to activate pyroptosis: the canonical pathway, the noncanonical pathway, and a new-found pathway. In the canonical pathway, some inflammasomes recruit and bind to apoptosis-associated speck-like protein containing a caspase recruitment domain (ASC), resulting in the formation of the ASC complex which recruits procaspase-1 and activates caspase-1. Caspase-1 is involved in the cleavage and maturation of proIL-18/1β, as well as the cleavage of gasderminD (GSDMD). The released N-terminal fragment of GSDMD (GSDMD-NT) causes pore formation in the plasma membrane, leading to secretion of IL-18/1β and water influx, which results in cell swelling and osmotic lysis (Liu et al., 2016; Fang et al., 2020). In the noncanonical pathway, bacterial-derived lipopolysaccharide (LPS) recognizes and activates caspase-4/5/11 to induce pyroptosis by cleaving GSDMD (Khanova et al., 2018; Rathinam et al., 2019). The new-found pathway is achieved by the cleavage of gasderminE (GSDME), which depends on the activation and participation of caspase-3 (Rogers et al., 2017; Wang et al., 2017). Pyroptosis appears to play a significant role in tumor progression and is linked to proliferation, migration, cell cycle, and treatment resistance in various of cancers, according to accumulated evidence (Heo et al., 2019; Yu et al., 2019; Tan et al., 2021). Recent studies have found that pyroptosis-related genes (PRGs) have satisfactory predictive abilities in the prognosis of PCa and could be used as novel tumor biomarkers (Fu et al., 2022; Hu et al., 2022; Wang et al., 2022). Meanwhile, its relationship with PCa immunity may provide assistance in the treatment of PCa (Li et al., 2022; Zhang et al., 2022). However, systematic evaluation of the relationship between differentially expressed PRGs and the prognosis, immune microenvironment, and treatment response of PCa is still worth further exploration.

Therefore, our study aims to develop a novel prognostic signature based on PRGs to systematically explore the relationships between the signature and clinicopathological characteristics and disease progression in PCa patients. In addition, we further investigated its correlation with the tumor microenvironment (TME), mutation profiles, and the patient’s response to immunotherapy and chemotherapy in PCa. This study provides new insights into the role of pyroptosis in PCa.

## Materials and methods

### Data collection and preprocessing

Gene expression data (FPKM value) for 495 prostate cancer samples and 52 normal samples were obtained from the TCGA official website (https://portal.gdc.cancer.gov/). The log2 transformation is used to normalize the TCGA-PRAD cohort. The clinical information for TCGA-PRAD was obtained from the cBioPortal for Cancer Genomics website (http://www.cbioportal.org/), as were the gene expression data and clinical information for the MSKCC/GSE21032 dataset. Patients who did not have survival information were excluded from our analysis. The clinical information of patients was shown in Supplementary Table S1. PRGs were gathered from the Molecular Signatures Database (MSigDB) (http://www.gsea-msigdb.org/gsea/msigdb/search.jsp) and previous reports (Liberzon et al., 2015; Wu et al., 2021a). We got a total gene set of 52 PRGs after deleting duplicate genes, found in Supplementary Table S2.

### The identification of prognostic hub genes

First, we used the R package “limma” to investigate the differential expression of PRGs between PCa tissues and adjacent nontumorous samples (Ritchie et al., 2015), and then we created a heat map with the R package “pheatmap” and a bar graph with the R packages “ggplot2” and “ggpubr” (Kolde, 2019). The “spearman” method was used to calculate the correlation coefficients of the differentially expressed pyroptosis-related genes (DE-PRGs) in PCa, and correlation plots were created using the R package “corrplot” (Wei and Simko, 2017). The STRING website (https://cn.string-db.org/) was used to calculate and generate the interaction network of DE-PRGs. Additionally, based on the DE-PRGs, we utilized the R package “ConsensusClusterPlus” for unsupervised clustering analysis of PCa samples (Wilkerson and Hayes, 2010), as well as the R package “survival” for survival analysis, to see whether the DE-PRGs were associated with patient differences (Therneau, 2020). For DE-PRGs, we utilized univariate Cox regression analysis to screen for genes associated with disease-free survival (DFS), and *p* < 0.05 was considered the cut-off value. LASSO regression was applied to lessen the risk of overfitting by R package “glmnet” (Simon et al., 2011). Finally, the multivariate stepwise Cox regression analysis was used to identify the hub genes, which were most associated with the prognosis of PCa.

### Validation of hub genes from RNA and protein expression levels

We obtained three PCa cell lines (LNCap, PC3, DU-145) cultured in RP1640 medium (Gibco) and one normal prostate epithelial cell line (RWPE-1) cultured in DMEM medium (Gibco) from the Second Hospital of Lanzhou University. Meanwhile all cells were cultured in a humidified incubator at 37°C and 5% CO2 with 10% fetal bovine serum added to every medium. Then we extracted the total RNA from the cells using TRIzol (AG21101; Hunan, China) reagent according to the manufacturer’s instructions, followed by reverse transcription. In addition, we measured the mRNA relative expression levels of the hub genes by real-time quantitative PCR, which were quantified by 2–ΔΔCT. The primer sequences of the hub genes and the internal reference gene could be found in Supplementary Table S3. Finally, we obtained immunohistochemistry (IHC) correlation data of hub genes from the Human Protein Atlas (HPA) (https://www.proteinatlas.org/) and further validated them by the protein expression levels of the genes (Uhlen et al., 2017).

### Construction and validation of the risk model

We utilized the training set (TCGA cohort) and the validation set (MSKCC cohort) to construct and validate the risk model, and both datasets calculated the risk score according to the formula: (expgene1 × coefgene1) + (expgene2 × coefgene2) + (expgene3 × coefgene3) +(expgene4 × coefgene4) +(expgene5 × coefgene5) +(expgene6 × coefgene6). The median risk score was the cut-off value to separate patients into high and low risk groups. Kaplan-Meier (KM) survival analysis with log-rank test and time-dependent subject work characteristics (ROC) analysis were used to assess the risk model’s correctness. We then utilized univariate and multivariate analyses to explore whether the risk score compared to clinicopathological characteristics of PCa was an independent prognostic factor. In addition, Wilcoxon and Kruskal-Wallis tests were used to examine the relationship between risk score and clinicopathological characteristics of PCa (age, T-stage, N-stage, Gleason score, and PSA value).

### Construction and validation of prognostic nomogram

Based on the independent prognostic factor risk score and Gleason score, we employed the R packages “rms” (Harrell, 2021) and “survival” (Therneau, 2020) to generate a nomogram to forecast the probability of DFS at 1, 3, and 5 years, and estimated the nomogram prediction scores for each patient. To evaluate the accuracy of the nomogram, we utilized the “calibration” function of the R package “rms” for calibration curve analysis and the R package “timeROC” for ROC analysis (Blanche et al., 2013; Harrell, 2021).

### Difference analysis in high and low risk groups, functional analysis

To better elucidate the biological function of FRGs in PCa, we obtained EDGs between high and low risk groups by the R package “limma” using *p*-value < 0.05 and log2 foldchange (log2FC) > 0.585 (Ritchie et al., 2015). The Gene Ontology (GO) enrichment and Kyoto Encyclopedia of Genes and Genomes (KEGG) pathway analyses were then carried out using the R packages “clusterProfiler” and “org.Hs.eg.DB,” with a critical value of *p* < 0.05 (Carlson et al., 2019; Wu et al., 2021b).

### Tumor microenvironment cell infiltration, tumor somatic mutation

The TME is comprised of tumor cells and non-tumor components such as blood vessels, immune cells, adipocytes, and tumor-associated fibroblasts (Binnewies et al., 2018). Hence, we analyzed the infiltration of immune cells in PCa samples by single sample gene set enrichment analysis (ssGSEA) using the R package “GSVA” (Hänzelmann et al., 2013) and the correlation of immune cells with risk score by the “Spearman” method using the R package “reshape2” (Wickham, 2007), and visualized by the R package “ggplot2” (Wickham, 2016). Then, we used the R package " estimate " to perform stromal score, immune score, and estimate score of PCa samples and to elucidate their relationship with high and low risk groups (Kosuke et al., 2016). The tumor mutation burden (TMB) data of PCa samples was collected from the TCGA database. The samples were separated into two groups based on the risk model, and the TMB score was computed using the R package “maftools” and displayed as a waterfall chart *via* the R package “ggplot2” (Wickham, 2016; Mayakonda et al., 2018). We also used the R package “reshape2″ to examine the link between risk score and TMB, followed by survival analysis using the R packages “survival” and “survminer” (Kassambara et al., 2021).

### Prediction of immunotherapy and drug sensitivity

The different expression of common immune checkpoint-related genes in high and low-risk score groups was achieved by the Wilcoxon test, and the “spearman” method was used to determine the correlation between immune checkpoint-related genes and risk score using the R package “reshape2” (Wickham, 2007), and visualized using the R package “ggplot2” (Wickham, 2016). In previous studies, the Immunophenoscore (IPS) was used to predict tumor response to immunotherapy with CTLA-4 and PD-1 blockers (Charoentong et al., 2017). Furthermore, we used the Wilcoxon test to compare IPS in high and low-risk groups after downloading IPS data for PCa from the Cancer Immunome Atlas (TCIA) (https://TCIA.at/home). In addition, the sensitivity of prostate cancer patients in high and low-risk groups to commonly used cell cycle chemotherapy drugs was computed using the R package “pRRophetic”, which was based on the Genomics of Drug Sensitivity in Cancer (GDSC, https://www.cancerrxgene.org) database (Geeleher et al., 2014).

### Statistical analysis

R software (version 4.1.2) and GraphPad Prism (version 9.0) were used for data analysis, statistics, and graphs in this study. The hub genes were discovered by univariate Cox regression, LASSO regression, and multivariate stepwise Cox regression analysis on DE-PRGs. The Wilcoxon test, Kruskal-Wallis test, and Dunnett’s test were used to compare differences between two or more groups as appropriate. The “Spearman” or “Pearson” approach was used to explore the relationship between distinct variables. The log-rank test of Kaplan-Meier analysis was used to perform the survival analysis. The above statistical methods produced significant results at *p* < 0.05.

## Results

### Expression and correlation of FRGs in TCGA-PRAD

**FIGURE 1:**
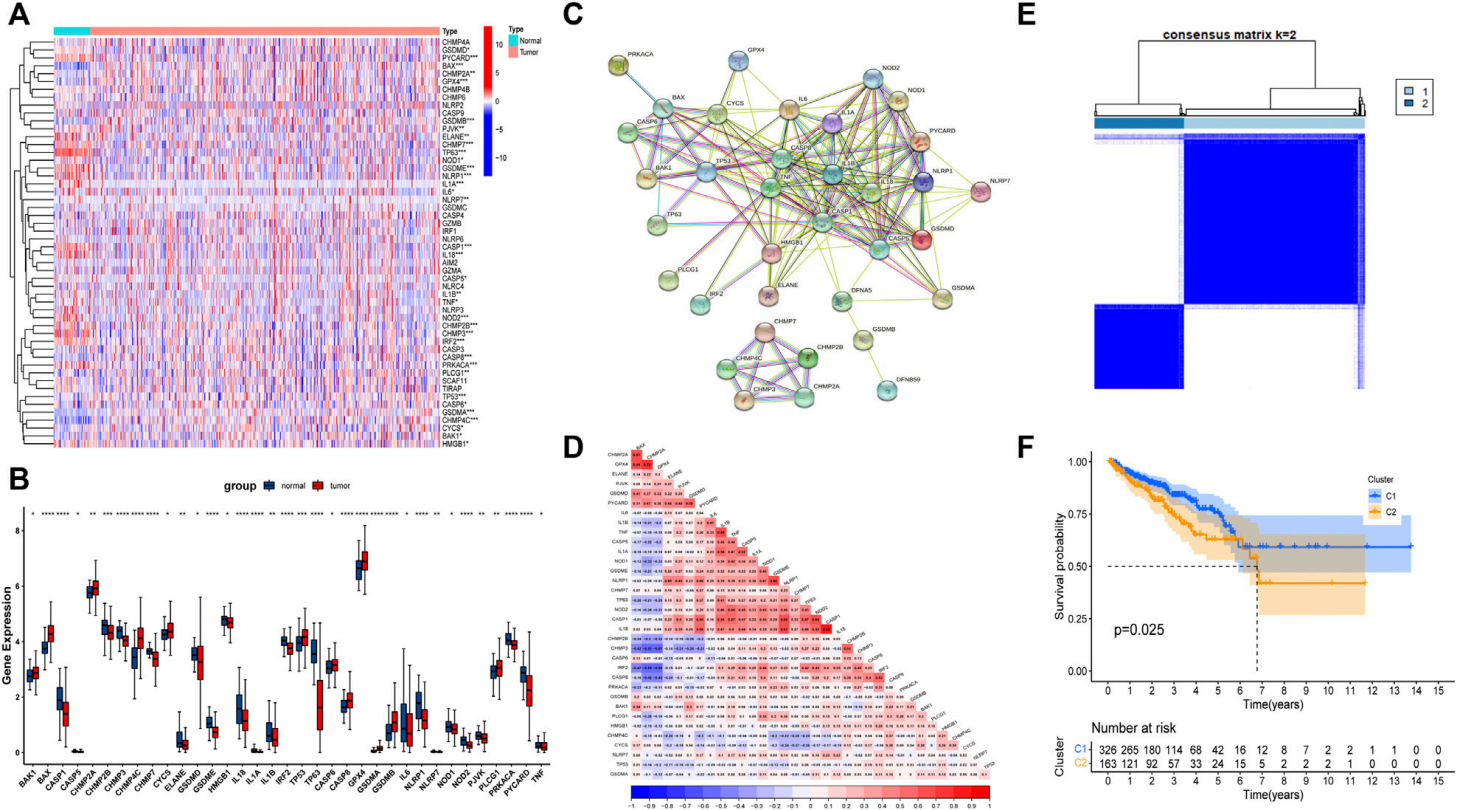
The landscape of expression and correlation of pyroptosis-related genes (PRGs) in prostate cancer. **(A)** The heatmap of 52 FRGs between prostate cancer tissues and normal prostate tissues. **(B)** The bar graph of 35 differentially expressed PRGs in prostate cancer and normal tissue. **(C)** The PPI network of 35 differentially expressed PRGs derived from the STRING database. **(D)** The correlation of 35 differentially expressed PRGs in prostate cancer. **(E)** Consensus Clustering matrix for k = 2. **(F)** The Kaplan-Meier (KM) curves of two clusters and cluster 2 had a worse DFS. *p < 0.05, **p < 0.01, ***p < 0.001, ****p < 0.0001.

First, we analyzed the expression of 52 FRGs in 495 tumor samples and 52 normal samples from the TCGA cohort, finding that 35 PRGs were expressed differently in normal and tumor tissues. From the heat map and boxplot, it can be seen that 12 genes, BAK1, CASP6, CYCS, PLCG1, TP53, CHMP2A, CASP8, GPX4, BAX, CHMP4C, GSDMB, and GSDMA, are highly expressed in tumor tissues, and the remaining 23 genes are highly expressed in normal tissues (Figures 1A,B). The PPI analysis revealed that these 35 PRGs had abundant interactions (Figure 1C). Meanwhile, the correlation analysis of these 35 genes in the TCGA cohort showed that they had a high correlation, such as GPX4 and CHMP2A (Figure 1D). Furthermore, we used these 35 genes to divide the TCGA cohort into two clusters (Figure 1E) and performed survival analysis, finding that patients in cluster 2 had a worse DFS (Figure 1F), implying a solid link between PRGs and patient differences. As a result, it was necessary for us to investigate the prognostic PRGs further.

### Construction and validation of PRGs prognostic model

**FIGURE 2:**
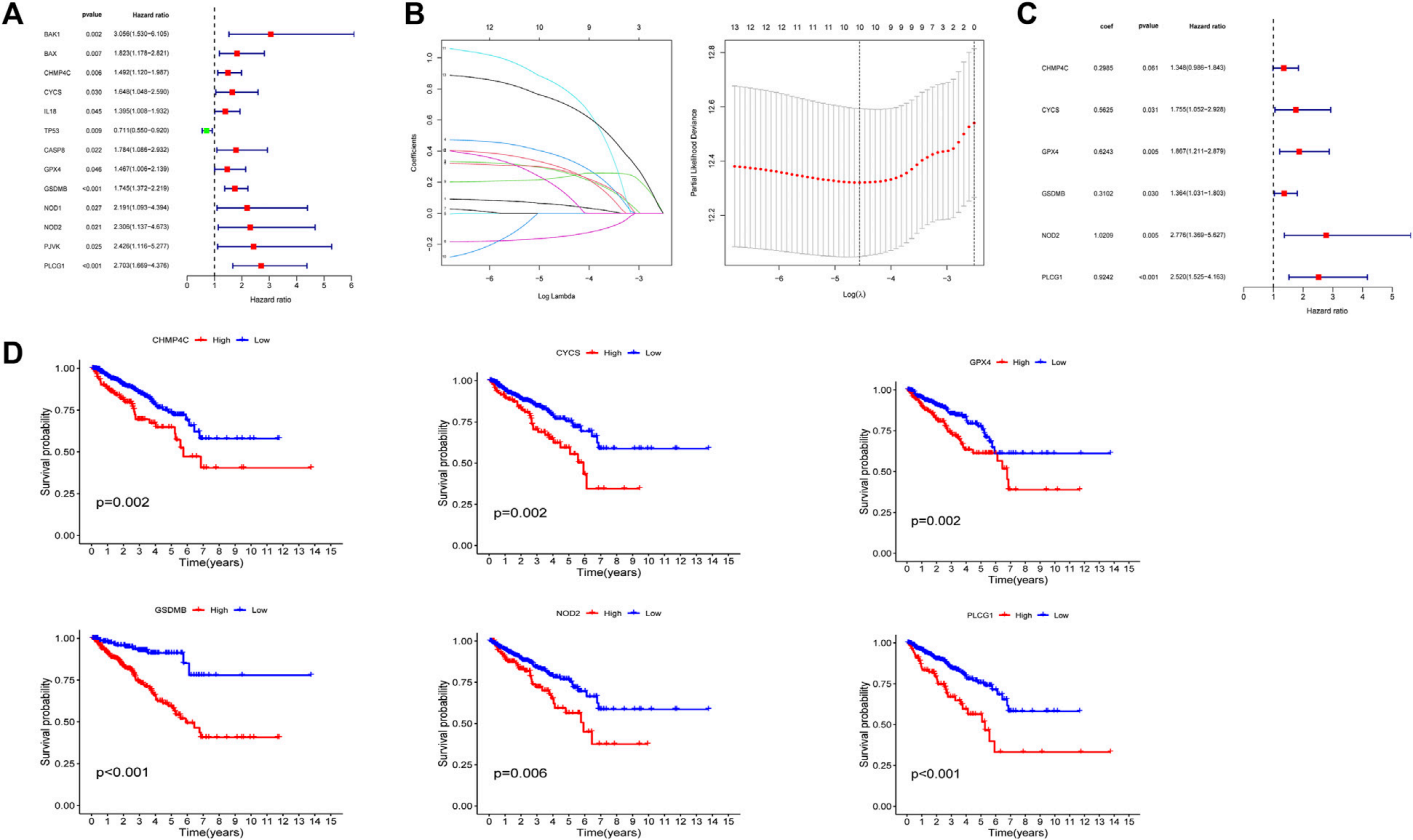
Identification and prognostic analysis of 6 hub genes. **(A)** 13 prognostic PRGs obtained by univariate Cox regression analysis. **(B)** LASSO analysis of 13 prognostic PRGs. **(C)** The risk coefficients for 6 hub PRGs obtained by multivariate Cox regression analysis. **(D)** The KM survival analysis of 6 hub genes.

**FIGURE 3:**
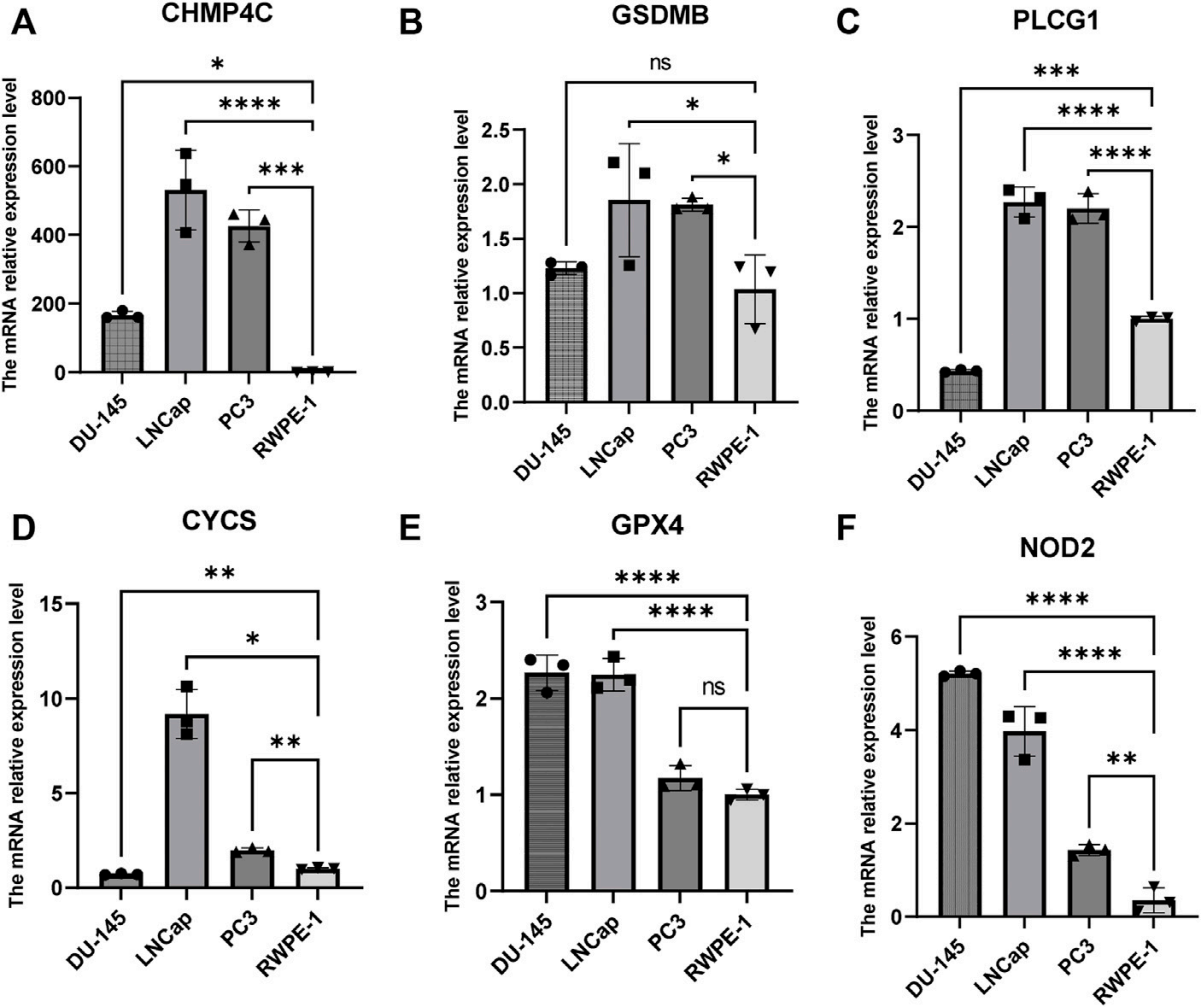
The mRNA expression levels of 6 hub genes in PCa cells (DU-145, LNCap, PC3) and the normal prostate epithelial cell line (RWPE-1). **(A–F)** The mRNA relative expression levels of CHMP4C, GSDMB, PLCG1, CYCS, GPX4, and NOD2 in DU145, LNCap, PC3, and RWPE -1.

**FIGURE 4:**
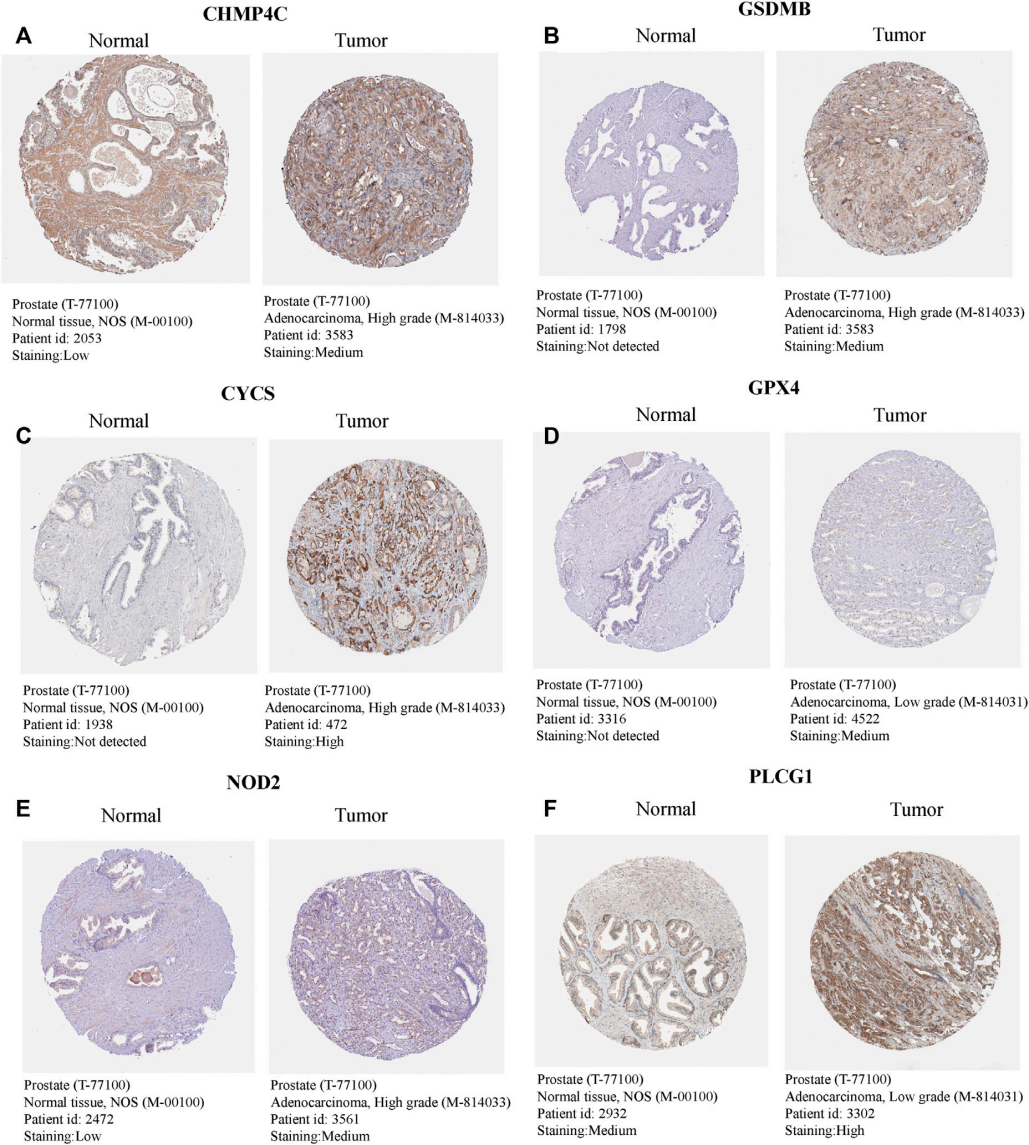
The expression levels of 6 hub genes in normal and tumour tissues. **(A–F)** The IHC - based protein expressions of CHMP4C, GSDMB, CYCS, GPX4, NOD2, and PLCG1 in PCa tissues and normal prostate tissues. These images were obtained from the HPA database.

**FIGURE 5:**
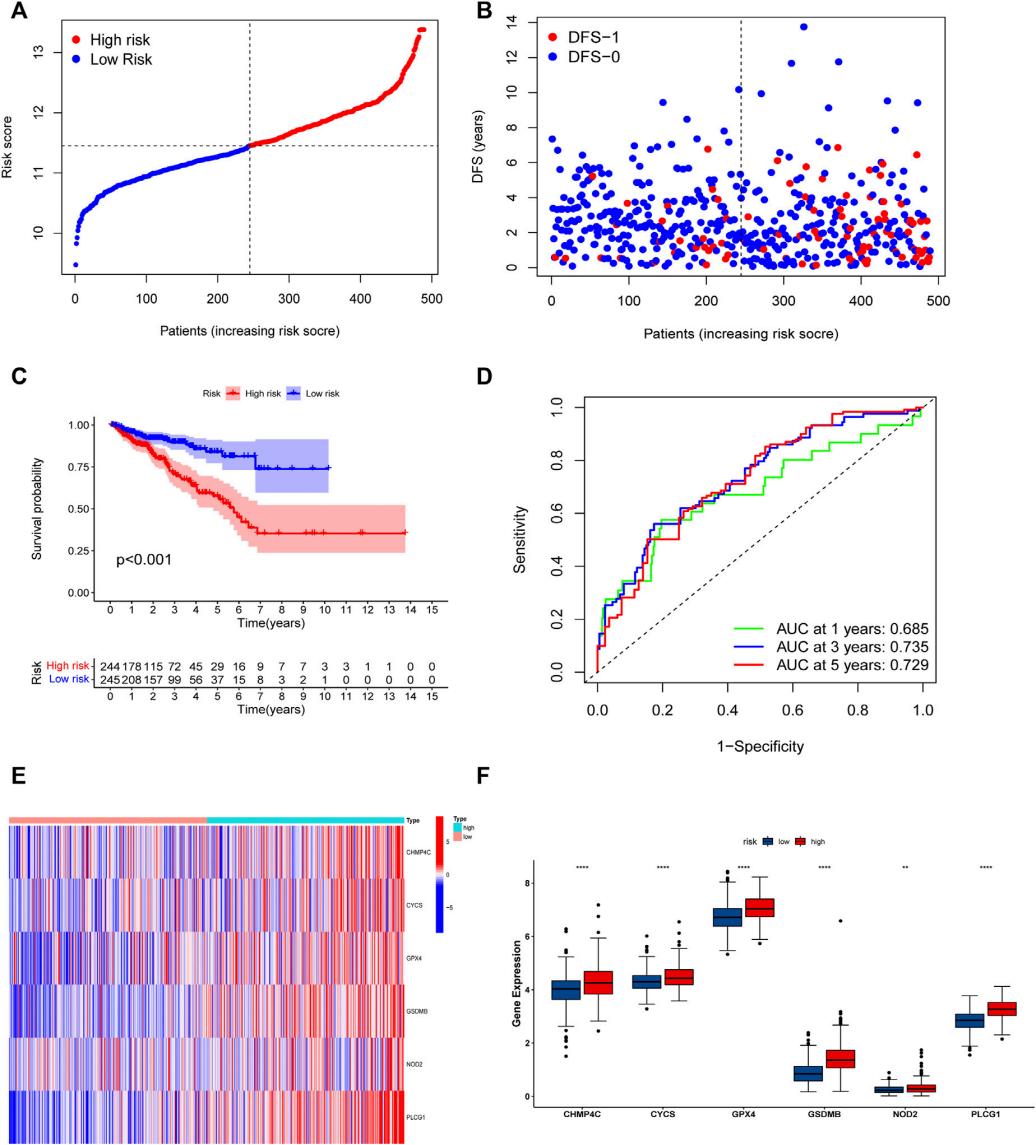
Risk model based on the 6 hub genes in TCGA cohort. **(A)** Distribution of patients’ risk scores. **(B)** Distribution of survival status of patients in high and low risk groups. **(C)** The KM curves in high and low risk groups of patients. **(D)** 1, 3, 5-year ROC curve. **(E,F)** The heatmap and bar graph of expression of 6 key genes in high and low risk groups.

**FIGURE 6:**
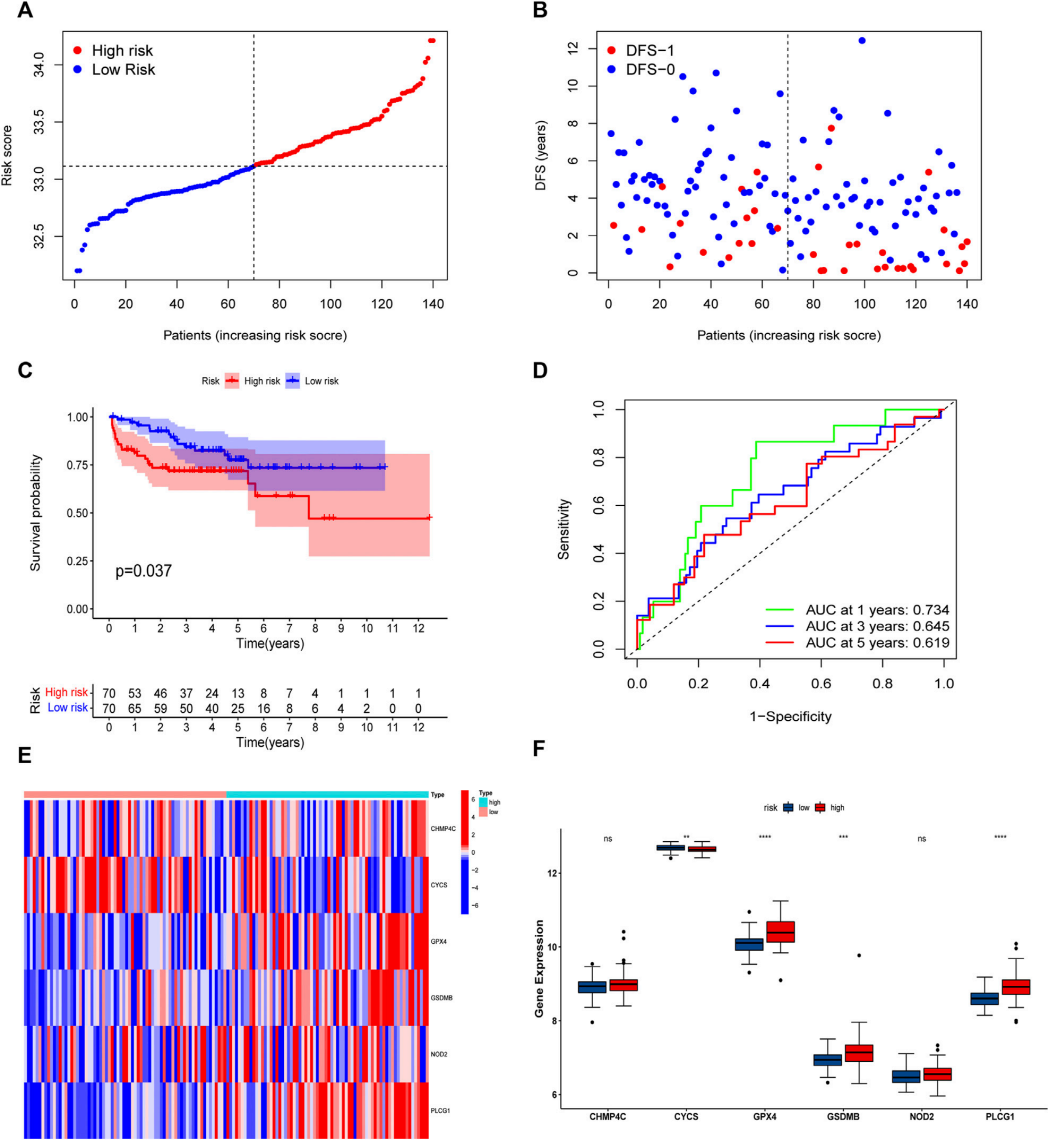
Risk model based on the 6 hub genes in MSKCC cohort. **(A)** Distribution of patients’ risk scores. **(B)** Distribution of survival status of patients in high and low risk groups. **(C)** The KM curves in high and low risk groups of patients. **(D)** 1, 3, 5-year ROC curves. **(E,F)** The heatmap and bar graph of expression of 6 key genes in high and low risk groups.

The univariate Cox regression analysis was used to analyze the above 35 DE-PRGs, and 13 genes were found to be associated with the DFS of PCa (Figure 2A). We then performed LASSO regression analysis with tenfold cross-validation on these 13 genes to mitigate the overfitting effect (Figure 2B). Subsequently, we performed multivariate stepwise Cox regression analysis to find 6 hub genes with the best prognostic value (Figure 2C). Before establishing the prognostic model, we conducted individual survival analyses on these 6 genes and discovered that genes with low expression had superior DFS (Figure 2D). In addition, we used qRT-PCR to compare the expression of hub genes (CHMP4C, GSDMB, NOD2, PLCG1, CYCS, GPX4) between prostate cancer cell lines (LNCap, PC3, DU-145) and the normal prostate epithelial cell line (RWPE-1). The results showed that, in comparison to RWPE-1, the 6 hub genes were generally more highly expressed in LNCaP, PC-3, and DU-145 cells (Figures 3A–F). Meanwhile, the IHC data obtained from HPA showed that the protein expression levels of the six genes were also higher in the prostate tumor tissues (Figures 4A–F). Therefore, we established a prognostic model using these six genes. Risk score = (0.2985 * expCHMP4C) + (0.5625 * expCYCS) + (0.6243 * expGPX4) + (0.3102 * expGSDMB) + (1.0209 * expNOD2) + (0.9242 * expPLCG1). Then, we divided PCa patients from the TCGA cohorts into high and low risk groups, with the median risk score as the cut-off value (Figure 5A). As shown in Figure 5B, PCa patients in the high-risk group had a higher likelihood of disease progression, which occurred earlier. According to the KM survival analysis, patients in the low-risk group had a better DFS than those in the high-risk group (Figure 5C). Furthermore, the area under the receiver operating characteristic curve (AUC) of the 1, 3, and 5-year DFS for the TCGA cohort was 0.685, 0.735, and 0.729 (Figure 5D), respectively, demonstrating that our risk models have a relatively high degree of accuracy. The heat map and box plot showed that all six hub genes had higher expression in the high-risk group of patients than in the low-risk group (Figures 5E,F). In the validation set (MSKCC cohort), the risk score was generated using the same formula, and PCa patients were classified into two groups: high and low risk, with the median risk score (Figure 6A). Although the expression of hub genes in the high and low-risk groups was slightly different from the TCGA cohort, the overall tendency was for the high-risk group to have higher gene expression (Figures 6E,F). Consistent with the results of the TCGA cohort, PCa patients in the high-risk group in the MSKCC cohort also had faster disease progression (Figure 6B). The results of KM survival analysis showed that patients in the low-risk group had a more favorable DFS (Figure 6C). And the AUC for 1, 3, and 5-year DFS were 0.734, 0.645, and 0.619, respectively (Figure 6D). These results indicated that the risk model in the MSKCC cohort could also play an important role in the prognosis of PCa.

### Independent prognostic value of risk models

**FIGURE 7:**
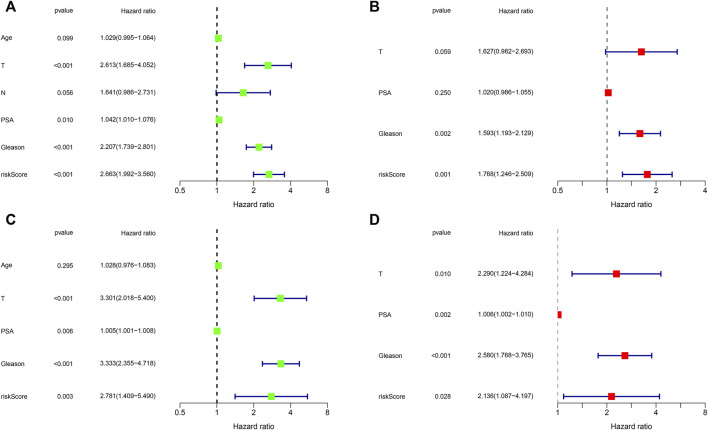
Independent prognostic value of risk score and clinical features. **(A,B)** Exploring independent prognostic factors using univariate **(A)** and multivariate **(B)** Cox regression analysis in TCGA cohort. **(C,D)** Validation of independent prognostic factors using univariate **(C)** and multivariate **(D)** COX regression analysis in MSKCC cohort.

**FIGURE 8:**
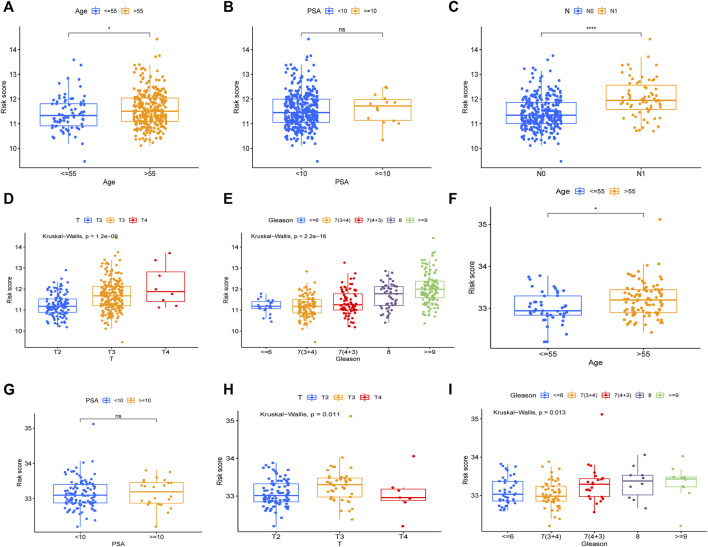
The relationship between risk score and clinical features of PCa. The relationship between Age **(A)**, PSA **(B)**, N **(C)**, T **(D)**, Gleason **(E)** and risk core in TCGA cohort. The relationship between Age **(F)**, PSA **(G)**, T **(H)**, Gleason **(I)** and risk score in MSKCC cohort.

To evaluate the prognostic significance of different clinical features of PCa patients and to evaluate whether the risk model can be used as an independent prognostic factor for PCa, we used univariate and multivariate Cox regression analyses on risk scores and different clinical features of PCa patients in the TCGA and MSKCC cohorts, respectively. In the TCGA cohort, the risk score and the Gleason score had *p < 0.05* (Figures 7A,B) in both univariate and multivariate analyses, indicating that they were both independent prognostic factors for PCa. Meanwhile, they got the same results in the validation set (MSKCC cohort) (Figures 7C,D). Furthermore, we evaluated the relationship between risk score and clinical features of PCa, finding that patients older than 55 years old had a higher risk score than patients younger than 55 years old, and that the risk score of patients increased as Gleason score, T-stage, and N-stage increased (Figures 8A–E). These results were also verified in the MSKCC cohort (Figures 8F–I).

### Construction and validation of the prognostic nomogram

**FIGURE 9:**
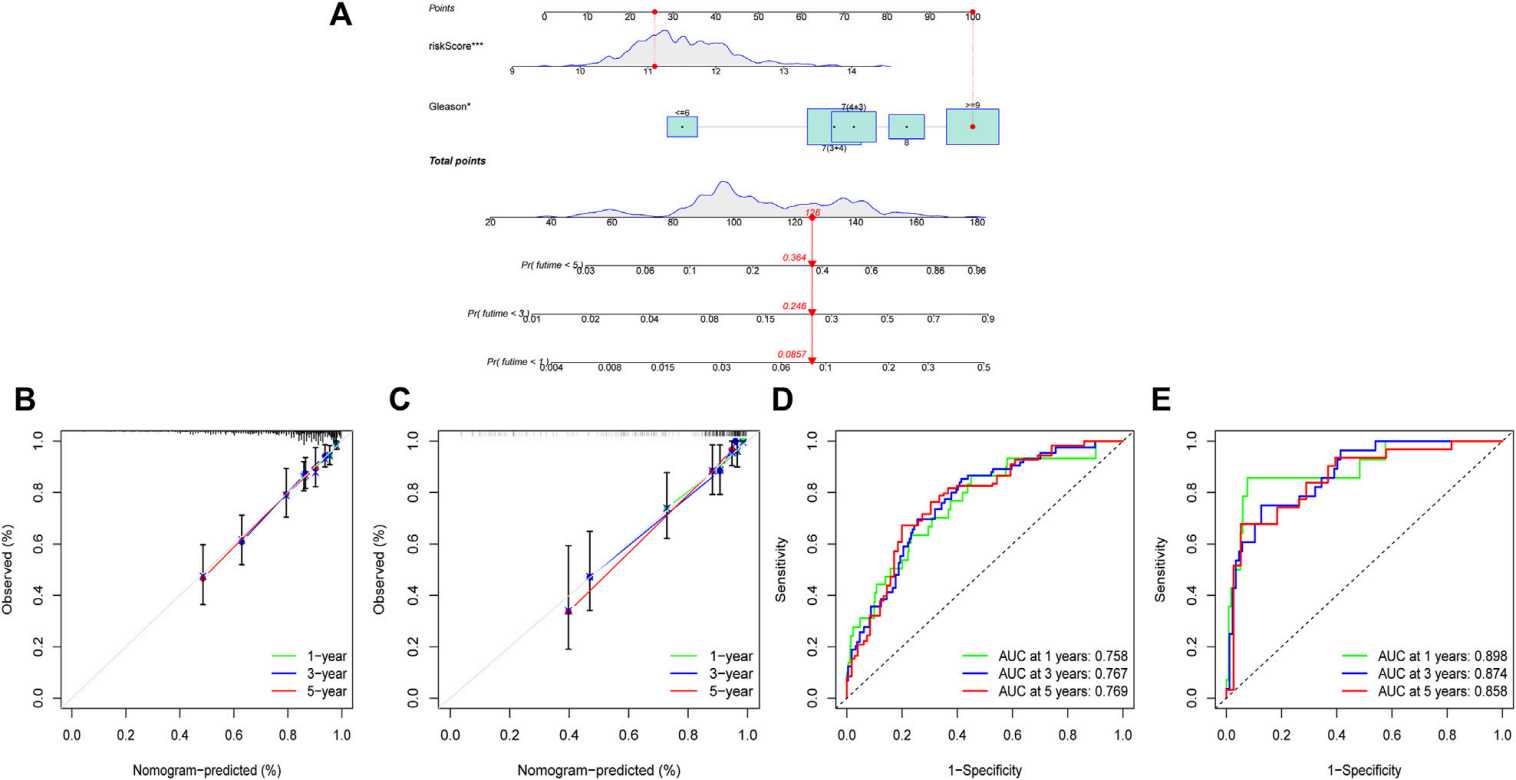
Construction and validation of a prognostic nomogram. **(A)** A nomogram established by the independent prognostic factors: risk score and Gleason. **(B)** The calibration curve of 1, 3, and 5-year in TCGA cohort. **(C)** The calibration curve of 1, 3, and 5-year in MSKCC cohort. **(D)** The ROC curve of 1, 3, 5-year in TCGA cohort. **(E)** The ROC curve of 1, 3, 5-year in MSKCC cohort.

We utilized the risk score and Gleason score to construct a prognosis nomogram based on the TCGA cohort because they are independent prognostic factors for PCa. The sample “TCGA-KK-A6E6” was chosen as an example simultaneously. The result showed that this patient’s probability of disease recurrence was 8.57%, 24.6%, and 36.4% at 1, 3, and 5 years, respectively (Figure 9A). Furthermore, the 1, 3, and 5-year calibration curves in the TCGA and MSKCC cohorts were all near the standard curve (Figures 9B,C). The AUC of the time-dependent ROC of the nomogram at 1, 3, and 5-year were all greater than 0.75 (Figures 9D,E), indicating that the prognostic nomogram we developed has high accuracy and validates its utility in predicting patient prognosis.

### Functional enrichment analysis

**FIGURE 10:**
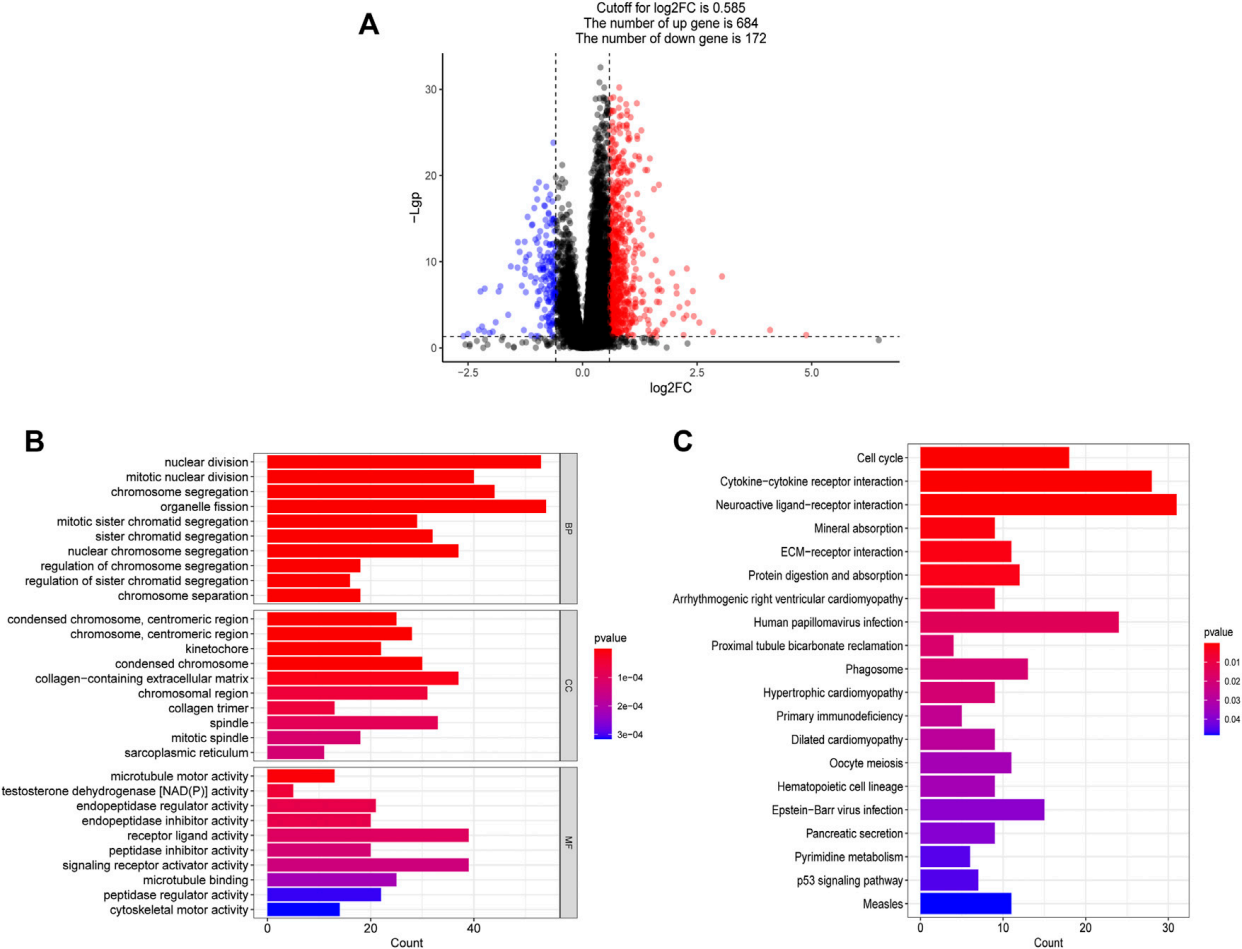
Functional analysis based on the DEGs between high and low risk groups. **(A)** The differentially expressed genes between high and low risk groups. **(B)** The bar graph of GO enrichment analysis, BP (biological process), CC (cellular component), MF (molecular function). **(C)** The bar graph of KEGG enrichment analysis.

The volcano diagram shows that there are 856 DEGs between high and low risk groups (FDR <0.05, |log2FC| ≥ 0.585), 684 of which are up-regulated genes and 172 of which are down-regulated genes (Figure 10A). The functional enrichment of these 856 genes was then performed using GO and KEGG enrichment analysis. Nuclear division, mitotic nuclear division, chromosomal segregation, mitotic sister chromatid segregation, and other cell cycle-related functions were mostly represented in the GO enrichment analysis (Figure 10B). In addition, KEGG enrichment analysis suggested that the genes were mainly associated with the cell cycle, cytokine-cytokine receptor interaction, ECM-receptor interaction, primary immunodeficiency. (Figure 10C).

### The characteristics of tumor microenvironment and tumor somatic mutation

**FIGURE 11:**
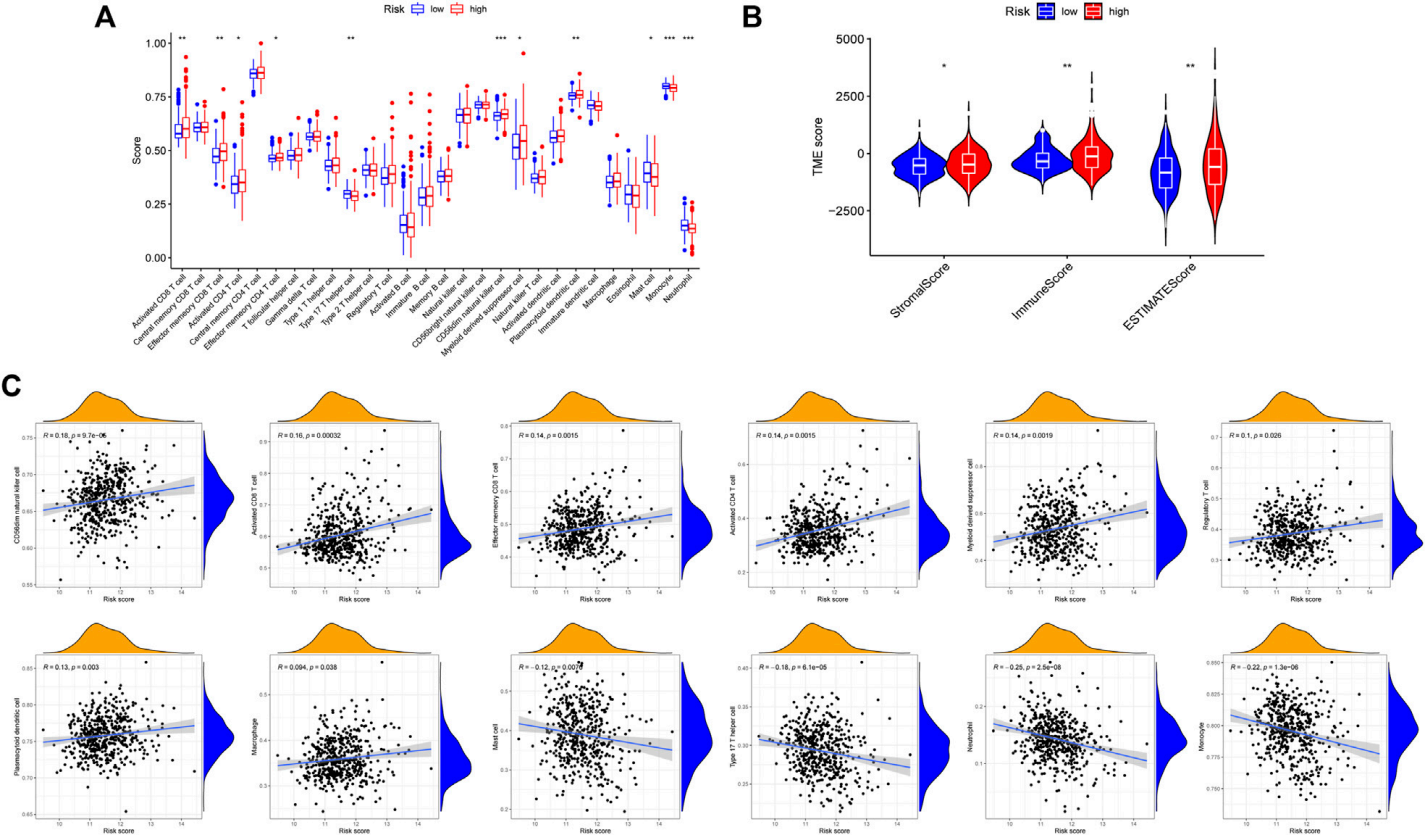
Comparison of immune microenvironment in high and low risk groups. **(A)** Differences in 28 immune cell infiltration between high and low risk groups by ssGSEA. **(B)** Differences in ImmuneScore, StromalScore and ESTIMATEScore between high and low risk groups. **(C)** Correlation between risk score and immune cells.

**FIGURE 12:**
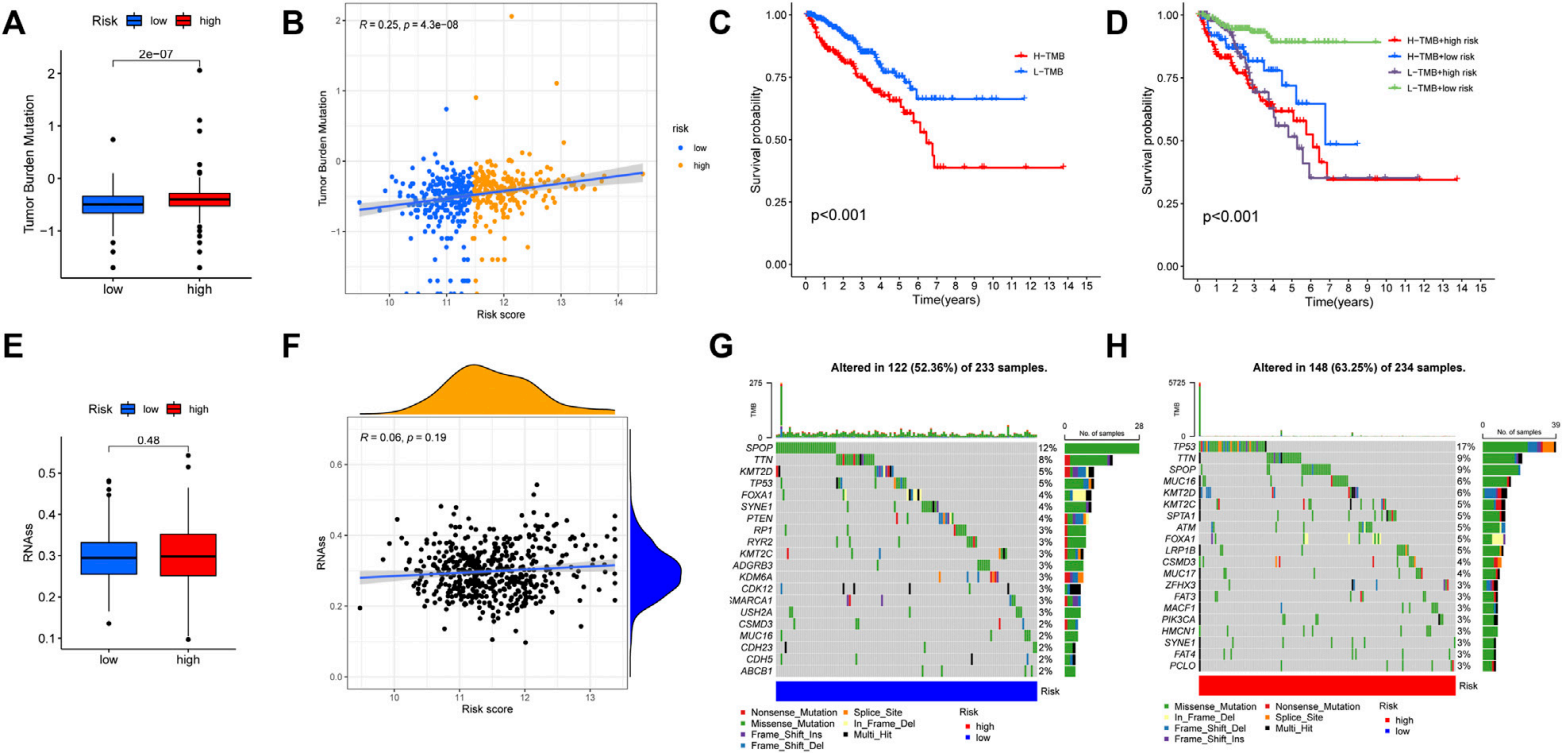
Comparison of tumor mutation burden (TMB) between high and low risk groups. **(A)** The TMB score was different between high and low risk groups. **(B)** The risk score was significantly and positively correlated with TMB. **(C)** The KM curves for patients with high and low TMB. **(D)** The KM curves showed that the L-TMB + low-risk group had the best prognosis. **(E,F)** No significant relationship was found between the risk score and mRNAsi. **(G)** The tumor somatic mutation of patients in the low-risk group. **(H)** The tumor somatic mutation of patients in the high-risk group.

According to the results of functional enrichment, the risk score was closely related to the cell cycle process, extracellular matrix, and cytokines. These factors play essential roles in the tumor microenvironment, tumor genetic alterations, and the treatment of tumors (Quail and Joyce, 2013). The following study discovered that the high-risk group had a larger infiltration of immune cells in the TCGA cohort (Figure 11A). The results of correlation analysis showed that the risk score was significantly positively correlated with the activated CD8 T cell, CD56dim natural killer cell, effector memory CD8 T cell, activated CD4 T cell, myeloid derived suppressor cell, regulatory T cell, plasmacytoid dendritic cell and macrophage. And the risk score was significantly negatively correlated with neutrophil, monocyte, mast cell and type 17 T helper cell (Figure 11C). Furthermore, the stromal score, immune score, and estimate score all exhibited higher expression in the high-risk group (Figure 11B), indicating that the tumor and non-tumor components of PCa in the high-risk group had a more complex relationship. We further analyzed the TMB of PCa, finding that there was a significant difference in TMB score between high and low-risk groups, with the high-risk group having the higher score (Figure 12A), and correlation analysis also revealed that risk score increased with increasing TMB score (Figure 12B).

Additionally, according to the optimal TMB threshold, PCa patients were separated into two groups: H-TMB and L-TMB, and the results of survival analysis revealed that patients in the low TMB group had a better DFS (Figure 12C). Similarly, when we combined the TMB and risk score groups, we found that the L-TMB + low-risk group had the best DFS (Figure 12D). Furthermore, no significant relationship was found between the risk score and mRNAsi (Figures 12E,F). Finally, there is a distinction between the high and low-risk groups in terms of the tumor somatic mutation. The overall mutation rate in the high-risk group is higher (63.25%) than in the low-risk group (52.36%). The mutation rate of “TP53” is highest in the high-risk group, while “SPOP” is highest in the low-risk group (Figures 12G,H).

### The sensitivity to immunotherapy and chemotherapy in the high and low risk groups

**FIGURE 13:**
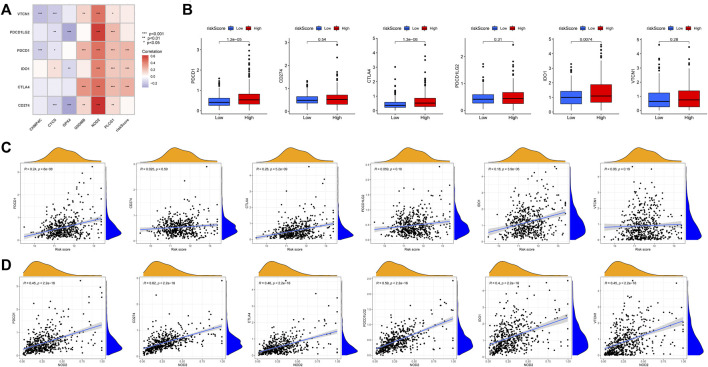
The relationship between immune checkpoint-related genes and risk score. **(A)** Heatmap of correlations between immune-check genes and central genes and risk score. **(B)** PD-1, CTLA4 and IDO1 were highly expressed in the high-risk group. The correlation between immune checkpoint-related genes and risk score**(C)** and NOD2**(D)**.

**FIGURE 14:**
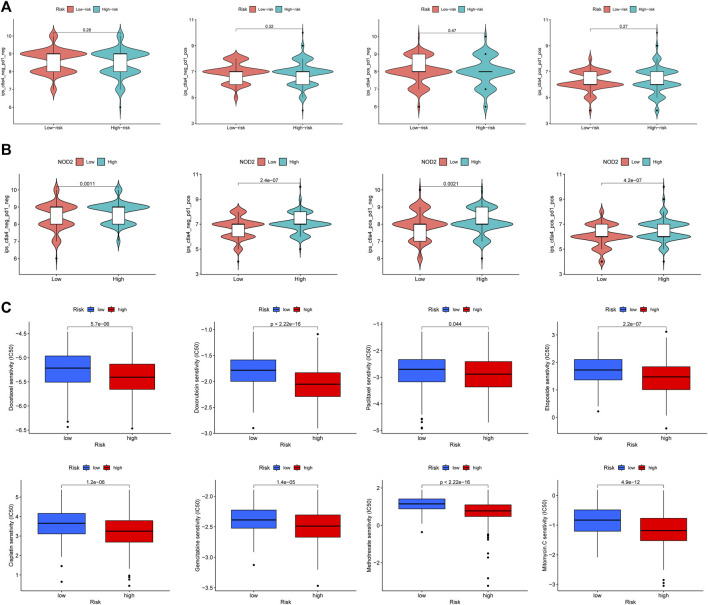
The responses to immunotherapy and chemotherapy. **(A)** The Violin plots of immunotherapy response between high and low risk groups. **(B)** The patients with high NOD2 expression were more sensitive to immunotherapy. **(C)** The estimated IC50 of common cell cycle-related chemotherapeutic agents between high and low risk groups.

Immunotherapy for tumors has entered a new era with the continuous development of immune checkpoint and chimeric antigen receptor (CAR) T cell therapies (Yang, 2015). The immune checkpoint blocking therapy was crucial in the immunotherapy of some malignancies (Grapin et al., 2019). We analyzed the association of PCa immune checkpoint-related genes PD-1 (PDCD1), PD-L1 (CD274), CTLA4, PD-L2 (PDCD1LG2), IDO1, and VTCN1 with the risk score and hub genes (Figure 13A) and discovered that PD-1, CTLA4, and IDO1 were highly expressed in the high-risk group (Figure 13B), and the risk score was significantly positively correlated with PD-1, CTLA4, and IDO1 (Figure 13C). Furthermore, Figure 13A showed that NOD2 was the hub gene with the strongest association to immune checkpoint-related genes, and NOD2 was significantly positively connected to these 6 genes (Figure 13D). Subsequently, we further downloaded IPS for PCa from the TCIA database. We analyzed the relationship between IPS and high and low-risk groups, finding that the four components of negative or positive responses for PD1 and CTLA4 were not significantly different in high and low-risk groups (Figure 14A). Fortunately, patients with high NOD2 expression had the higher IPS than those with low expression (Figure 14B).

The risk score was closely related to the cell cycle progression of PCa according to the above functional enrichment analysis, and we further analyzed the response of PCa patients in the TCGA cohort to eight common cell cycle-related chemotherapy drugs (Docetaxel, Gemcitabine, Paclitaxel, Doxorubicin, Cisplatin, Etoposide, Mitomycin, and Methotrexate). The results revealed that these drugs had lower half maximal (50%) inhibitory concentration (IC50) in patients of the high-risk group (Figure 14C), implying that these patients may be more sensitive to these drugs.

## Discussion

PCa is a common male urological malignancy. In Asia, the 5-year survival rate for PCa is above 60% (Hassanipour et al., 2020). Between 2001 and 2016 in the United States, the 10-year survival rate for localized stage PCa approached 100% (Siegel et al., 2020). However, a large proportion of PCa patients might experience disease progression, even to the CRPC stage, which increases the risk of PCa-specific death. From 2011 to 2016, the 5-year survival rate for distant stage PCa in the United States was only 32.3% (Siegel et al., 2020). Therefore, there is an urgent need to identify novel prognostic signatures for PCa to improve precise treatment and health management.

Pyroptosis, a new type of programmed cell death that involves the release of inflammatory factors and some immunological responses, is closely related to the occurrence and development of tumors (Du et al., 2021). At present, much research has explored the role of pyroptosis in various tumors, establishing some effective models for predicting prognosis and treatment response and analyzing the potential role of pyroptosis in the tumor microenvironment (Wu et al., 2021a; Li et al., 2021; Shao et al., 2021). A recent study explored the correlation between pyroptosis and PCa patients, resulting in a new signature for predicting PCa patients’ prognosis (Hu et al., 2022). However, the relationship between the members of PRGs and PCa still remains worthy of research.

In this study, we first obtained 35 PRGs that were differentially expressed between tumor and normal tissues in the TCGA-PRAD cohort. Following that, six hub genes (CHMP4C, NOD2, GSDMB, PLCG1, GPX4, CYCS) were found to be strongly associated with the DFS of PCa using univariate cox regression, LASSO regression, and multivariate stepwise Cox regression analysis. In the TCGA-PRAD cohort, we established a risk model of PRGs using these six hub genes, and patients were separated into high and low-risk groups based on their median risk score, with the patients in the high-risk group being found to be more likely to experience a worse DFS. These findings were validated in the MSKCC external validation dataset.

According to previous research, these hub genes are closely related to the occurrence and development of various diseases. CHMP4C, an ESCRT-III subunit, is involved in the abscission checkpoint (NoCut) in response to mitotic problems. Dysregulation of abscission by CHMP4C may act in concert with oncogene-induced mitotic stress to promote genomic instability and tumorigenesis (Sadler et al., 2018). It has been reported that CHMP4C may play an important role in aggressive prostate cancer and may be a potential therapeutic target (Fujita et al., 2017). NOD2 is an intracellular pattern recognition receptor that senses bacterial peptidoglycan conserved motifs in the cytosol and stimulates the host immune response (Ferrand et al., 2019). It has been reported that NOD2 has been linked to the innate immune response of prostate epithelial cells and the occurrence and progression of prostate cancer (Kang et al., 2012). GSDMB, a member of the Gasdermin family, is a downstream effector protein in the pyroptosis pathway (Li et al., 2020), and it has been related to the development of bladder and stomach malignancies in multiple studies (Zhou et al., 2020; He et al., 2021). PLCG1 is a member of the phosphatidylinositol-specific phospholipase C (PLC) family that hydrolyzes phosphatidylinositol 4,5-bisphosphate (PIP2) to generate inositol 1,4,5-trisphosphate and diacylglycerol (DAG), which is associated with the proliferation and invasion of tumor cells. Aberrant expression and regulation of PLCG1 have been linked to the development of various cancers, including breast, lung, pancreatic, gastric, prostate, and ovarian cancers (Mandal et al., 2021). GPX4 is an enzyme that explicitly reduces phospholipid hydroperoxides to repair oxidative lipid damage (Gaschler and Stockwell, 2017). GPX4 is not only a negative regulator of ferroptosis and has been associated with numerous cancers (Riegman et al., 2020), but it also helps to attenuate lipid peroxidation, inflammasome activation, and pyroptosis in the context of sepsis (Kang et al., 2018). CYCS, or cytochrome c, has been implicated in numerous regulated cell death forms in addition to being an electron carrier in the mitochondrial respiratory chain (Bock and Tait, 2020), such as the release of cytochrome c into the cytoplasmic matrix upon stimulation by Bax to activate caspase-3, which leads to pyroptosis by triggering GSDME cleavage (Zhou et al., 2018). Meanwhile, a previous study indicated that cytochrome c may impact the sensitivity of the PCa cell line (PC3) to chemotherapeutic agents (Grayson et al., 2021). Therefore, these hub genes might be potential therapeutic targets for PCa.

The following study investigated the association between risk score and clinicopathological characteristics, and discovered that the high-risk group had a higher degree of malignancy. Meanwhile, in our study, only the risk score and the Gleason score were independent prognostic factors for PCa, showing that the risk model had a strong prognostic value. Furthermore, a nomogram with two independent prognostic factors can assist clinicians in predicting patient prognosis and provide a more trustworthy reference for health management than a single routine clinical parameter.

To explore the functional mechanisms of the risk model, we first obtained 856 differentially expressed genes between the high and low-risk groups, and functional enrichment analysis revealed that these genes were mainly closely related to cell cycle processes. And there were several cell cycle related drugs in chemotherapy for PCa, such as Docetaxel, Gemcitabine, Paclitaxel, Doxorubicin, Cisplatin, Etoposide, Mitomycin, and Methotrexate. We then calculated their estimated IC50s in different patients. The estimated IC50s of these drugs were all lower in the high-risk group than in the low-risk group, indicating that patients in the high-risk group were more sensitive to these drugs.

There is mounting evidence that cell cycle processes are not only linked to tumor development (Liu et al., 2022), but also play a role in immune escape and immunotherapy (Bednarski and Sleckman, 2019). In the subsequent study, we discovered that the high-risk group had more immune cell expression than the low-risk group, and a majority of the immune cell infiltration was positively correlated with the risk score, suggesting that there may be more abundant immune effects in the high-risk group. Later, immunotherapy-related markers such as TMB, mRNAsi, and IPS were incorporated into further studies. The results showed that the risk score was positively correlated with the TMB score, and the total somatic mutation rate in the high-risk group (63.25%) was higher than that in the low-risk group (52.36%). However, there was no obvious link between risk scores and mRNAsi. In addition, although the immune checkpoint-related genes PD-1 and CTLA4 were significantly higher expression in the high-risk group, the IPS analysis revealed no significant difference in the response of patients in the high and low-risk groups to PD1 and CTLA4 immune checkpoint inhibitors. Fortunately, the hub gene NOD2 was significantly and positively correlated with the expression of immune checkpoint-related genes, and patients with high NOD2 expression also had the higher IPS than those with low expression. These results point to a complex relationship between the PRGs and the immune microenvironment of PCa, which could be helpful for future research into PCa immunotherapy, particularly the function of the hub genes.

We constructed a risk model of PCa using PRGs and analyzed the relationship between the risk model and PCa from multiple perspectives, which may have good clinical significance. However, our study also has certain limitations. The sample size from the TCGA and MSKCC databases may not be sufficient and more data needs to be collected. At the same time, further *in vitro* experimental research and clinical trials are required to confirm our findings.

## Conclusion

In conclusion, our study demonstrates that pyroptosis plays a vital role in PCa prognosis and that pyroptosis has some effects on the regulation of the TME in PCa. Meanwhile, we provide new insights into PCa prognostic research and assist in developing more effective individual treatment strategies.

## Data Availability

The datasets presented in this study can be found in online repositories. The names of the repository/repositories and accession number(s) can be found in the article/Supplementary Material.
