# Supplementary material for: A novel signature based on pyroptosis-related genes for predicting prognosis and treatment response in prostate cancer patients
Source: Front Genet. 2022 Oct 27;13:1006151. doi: 10.3389/fgene.2022.1006151 (PMC9648539; doi:10.3389/fgene.2022.1006151)
Supplement: Supplementary file 1 [file DataSheet1.docx]

Supplementary Tables

Supplementary Table 1: the clinical information of patients.

| Variables | Type | TCGA  (n=495) | MSKCC  (n=140) |
| --- | --- | --- | --- |
| Age | <=55  >55 | 110 (22.2%)  385 (77.8%) | 46 (32.9%)  94 (67.1%) |
| T | T2  T3  T4  unknow | 187 (37.8%)  291 (58.8%)  10 (2.0%)  7 (1.4%) | 86 (61.4%)  47 (33.6%)  7 (5.0%)  - |
| N | N0  N1  unknow | 344 (69.5%)  78 (15.8%)  73 (14.7%) | -  -  - |
| PSA | <10  >=10  unknow | 422 (85.3%)  16 (3.2%)  57 (11.5%) | 114 (81.4%)  24 (17.1%)  2 (1.4%) |
| Gleason score | <=6  7(3+4)  7(4+3)  8  >=9  unknow | 46 (9.3%)  144 (29.1%)  101 (20.4%)  63 (12.7%)  141 (28.5%)  - | 41 (29.3%)  53 (37.9%)  23 (16.4%)  11 (7.9%)  10 (7.1%)  2 (1.4%) |

Supplementary Table 2: the 52 pyroptosis-related genes.

| Genes | Full name |
| --- | --- |
| AIM2 | Absent in melanoma 2 |
| BAK1 | BRI1-associated receptor kinase 1 |
| BAX | Bcl-2-associated X protein |
| CASP1 | Cysteine-aspartic acid protease-1 |
| CASP3 | Cysteine-aspartic acid protease-3 |
| CASP4 | Cysteine-aspartic acid protease-4 |
| CASP5 | Cysteine-aspartic acid protease-5 |
| CASP6 | Cysteine-aspartic acid protease-6 |
| CASP8 | Cysteine-aspartic acid protease-8 |
| CASP9 | Cysteine-aspartic acid protease-9 |
| CHMP2A | Charged Multivesicular Body Protein 2A |
| CHMP2B | Charged Multivesicular Body Protein 2B |
|  |  |
| CHMP3 | Charged Multivesicular Body Protein 3 |
| CHMP4A | Charged Multivesicular Body Protein 4A |
| CHMP4B | Charged Multivesicular Body Protein 4B |
| CHMP4C | Charged Multivesicular Body Protein 4C |
| CHMP6 | Charged Multivesicular Body Protein 6 |
| CHMP7 | Charged Multivesicular Body Protein 7 |
| CYCS | Recombinant Cytochrome C, Somatic |
| ELANE | Elastase, neutrophil expressed |
| GPX4 | Glutathione peroxidase 4 |
| GSDMA | Gasdermin A |
| GSDMB | Gasdermin B |
| GSDMC | Gasdermin C |
| GSDMD | Gasdermin D |
| GSDME | Gasdermin E |
| GZMA | Granzyme A |
| GZMB | Granzyme B |
| HMGB1 | High-mobility group box-1 protein |
| IL18 | Interleukin 18 |
| IL1A | Interleukin 1 a |
| IL1B | Interleukin 1 beta |
| IL6 | Interleukin 6 |
| IRF1 | Interferon regulatory factor 1 |
| IRF2 | Interferon regulatory factor 2 |
| NLRC4 | NLR family CARD domain containing 4 |
| NLRP1 | NLR family pyrin domain containing 1 |
| NLRP2 | NLR family pyrin domain containing 2 |
| NLRP3 | NLR family pyrin domain containing 3 |
| NLRP6 | NLR family pyrin domain containing 6 |
| NLRP7 | NLR family pyrin domain containing 7 |
| NOD1 | Nucleotide binding oligomerization domain containing 1 |
| NOD2 | Nucleotide binding oligomerization domain containing 2 |
| PJVK | Pejvakin/deafness, autosomal recessive 59 |
| PLCG1 | Phospholipase C gamma 1 |
| PRKACA | Protein kinase camp-activated catalytic subunit alpha |
| PYCARD | PYD and CARD domain containing |
| SCAF11 | SR-related CTD associated factor 11 |
| TIRAP | TIR domain containing adaptor protein |
| TNF | Tumor necrosis factor |
| TP53 | Tumor Protein P53 |
| TP63 | Tumor Protein P63 |

Supplementary Table 3: the sequences of all the primers

| Primer | Sequence (5'to3') |
| --- | --- |
| CHMP4C-F | GCAGCAAAAGCGATGAAATCT |
| CHMP4C-R | ATTTCTTGGGCGATATCCTGT |
| GSDMB-F | TAAACTCCCTCGCTAAGTGCC |
| GSDMB-R | ATGTGTAGCTCCCCGGAAATC |
| NOD2-F | ATGAAATCAGGTTGCCGATCT |
| NOD2-R | CAATCCATTCGCTTTCACCGT |
| GPX4-F | TCGACCTGCACGCCCGATAC |
| GPX4-R | CTGGCTCCTGCTTCCCGAACT |
| PLCG1-F | GCCGGACCAGTCACATTGCTT |
| PLCG1-R | AGCCCTTGATCCACATGTTCACT |
| CYCS-F | TTATGAAGTGTTCCCAGTGCC |
| CYCS-R | TTCCGCCCAAAGAGACCATG |
| ACTB-F | CCAACCGCGAGAAGATGACC |
| ACTB-R | AGCACAGCCTGGATAGCAAC |

F: forward R: reverse
